# Miscellaney

**Published:** 1897-02

**Authors:** 


### Miscellany.

### VIVISECTION IN THE DISTRICT OF COLUMBIA.

AT THE Richmond meeting, September, 1896, of the American Association of Obstetricians and Gynecologists, Dr. Thomas E. McArdle, of Washington, D. C., offered the following preamble and resolutions:

The American Association of Obstetricians and Gynecologists, assembled in annual session at Richmond, Va., September 22 to 24, 1896, desires to present to the Congress of the United States a protest against the passage of Senate Bill 1552.

WHEREAS, The enactment into law of the specified bill would greatly interfere with and retard the investigations that are at present being conducted at Washington by the laboratories connected with the Marine Hospital, the offices of the Surgeon-General of the United States Army and Navy, and the Bureau of Animal Industry of the Department of Agriculture; and

WHEREAS, The results of their investigations have been of immense importance to the health and wealth of the people of the country; and

WHEREAS, More brilliant results are promised for the near future in connection with preventive medicine and the health of men and animals; therefore, be it

*Resolved*, That this Association protests against the proposed legislation by Congress, which has for its object the restriction of animal experimentation in the District of Columbia; and, while opposing needless cruelty upon animals in the public schools, this Association considers that those who are trained in the special line of research necessary for the conduct of the work referred to are the persons best able to decide upon the advisability and utility of animal experimentation, and should not be hindered in the prosecution of their humane work. Be it further

*Resolved*, That a copy of these resolutions be sent to the members of the House and Senate of the United States Congress and also to the President of the United States.

The resolutions were unanimously adopted.

The Robinson Thermal Bath Cabinet, which is advertised in our columns, commends itself to everyone who needs hot air or medicated baths. It is a simple, effectual and economical method of administering thermal baths for many diseases, and when used under the advice of a competent physician cannot fail to give satisfaction. The attention of physicians is invited to this subject and the Robinson Thermal Bath Company is anxious to receive calls from physicians and to furnish any information desired.

